# Paradoxical inflation of prediction model performance measures in minority patient subgroups: the role of upstream bias

**DOI:** 10.1038/s44401-026-00158-0

**Published:** 2026-10-21

**Authors:** Rahul Ladhania, Allister Z. Ho, Paramveer Dhillon, Chad M. Brummett, Karandeep Singh, Anne C. Fernandez

**Affiliations:** 1 Department of Health Management & Policy, School of Public Health, University of Michigan, Ann Arbor, MI USA; 2 Department of Biostatistics, School of Public Health, University of Michigan, Ann Arbor, MI USA; 3 Institute for Healthcare Policy and Innovation, University of Michigan, Ann Arbor, MI USA; 4 Department of Bioinformatics, University of Michigan Medical School, Ann Arbor, MI USA; 5 School of Information, University of Michigan, Ann Arbor, MI USA; 6 Division of Pain Medicine, Department of Anesthesiology, University of Michigan Medical School, Ann Arbor, MI USA; 7 Opioid Research Institute, University of Michigan, Ann Arbor, MI USA; 8 Joan and Irwin Jacobs Center for Health Innovation, University of California, San Diego, CA USA; 9 Division of Biomedical Informatics, Department of Medicine, University of California, San Diego, CA USA; 10 Department of Psychiatry, University of Michigan Medical School, Ann Arbor, MI USA

**Keywords:** Health care, Medical research

## Abstract

Predictive models in healthcare AI are expected to perform worse in underrepresented subgroups due to upstream biases such as underrepresentation, differential care access, and potential measurement error. In a previously published model predicting persistent postoperative opioid use, we surprisingly observed substantially higher model performance in Black patients (which comprised less than 5% of the sample) than in White patients. In this study, we used subgroup matching to investigate whether differences in patient characteristics contributed to the observed disparity, and replicate our findings in a second independent model. Across both models, performance differences - measured by AUC and other performance metrics - were attenuated after matching, suggesting that upstream biases may paradoxically inflate apparent model performance in minoritized patient groups. These findings challenge conventional assumptions about bias and highlight the importance of investigating performance differences using subgroup-level covariate alignment. Our results emphasize the need for careful evaluation of subgroup model metrics to ensure fair and trustworthy deployment of clinical AI.

## Introduction

Several national and international AI guidelines emphasize the need for predictive health AI models to be fair^1–4^, and many major AI vendors provide tools to assess fairness^5–8^. The need to consider fairness is especially critical when these models are used to allocate healthcare resources because biased allocation of resources can worsen inequities^9^. A model is considered fair when it performs comparably across subgroups and equitably allocates resources based on patients’ risk^10–13^. As a consequence, efforts to evaluate and mitigate bias often focus on first measuring performance across subgroups, followed by assessing how resource allocations would vary by group^8^. This approach has been successful in uncovering specific types of unfairness, including models used to prioritize patients for chronic care management or pain treatment^9,14^.

Recently, there has been growing recognition of the role of upstream bias in producing unfair models. Upstream biases are those introduced into data before AI models are trained^15^. These can result from differences in care, differences in technology, underrepresentation, and inaccurate or inconsistent measurement within subgroups of people^16,17^. Correcting these deficiencies can improve model performance within subgroups^18^, highlighting the importance of recognizing and correcting upstream biases.

Upstream biases are generally expected to produce worse performance among affected subgroups, particularly when those groups are underrepresented in training data or when systemic disparities distort the data generating process^19,20^. Given the typically limited sample sizes of Black and other minority patients in many healthcare datasets, this often leads to reduced model performance for these groups^20^. It was therefore *surprising* when a recent opioid prediction model developed by our team using the Michigan Genomics Initiative (MGI) perioperative cohort, hereafter the MGI model, produced better performance in Black patients by a substantial margin^21^. In this prior work, the area under the receiver operating characteristic curve (AUC) for a model predicting persistent opioid use (POU) after surgery was observed to be 0.93 (95% CI 0.88–0.97) in Black patients and 0.86 (95% CI 0.85–0.88) in White patients (*p* = 0.004). While the higher AUC in Black patients would typically be reassuring, this result was discordant with prior evidence, which has shown that Black patients are less likely than White patients to receive optimal treatment, including worse access to opioids^22,23^.

In this study, we sought to understand whether upstream bias could counterintuitively produce better performance in the affected subgroup. We did this by first evaluating the robustness of our prior work’s findings by replicating them using another published opioid prediction model, hereafter the refit claims model^24^, which predicts new POU after surgery among opioid-naive patients. Upon finding a similar result (higher AUC in Black patients), we used two types of subgroup matching to explore the relationship between baseline characteristics and observed model performance. This approach provides a systematic way to evaluate the presence and meaning of observed performance differences across subgroups. We hypothesized that observed performance differences were linked to differences in patient characteristics among subgroups in our study cohort, an indicator of potential upstream bias.

## Results

This section first describes the study cohorts used to develop each model, then evaluates whether the previously reported higher AUC in Black patients replicates in an independently developed model. We then compare baseline characteristics between Black and White patients to identify factors that may explain this performance difference, and use subgroup matching to test whether these characteristics account for the observed gap.

### Study cohort

For the MGI model, the cohort included 24,040 patients, with a median age of 55, with 53% female, 48% opioid-naive prior to surgery, and predominantly non-Hispanic White (89%). (Table 1A). 20% of patients experienced postoperative POU. Patients without POU were more likely to be opioid-naive at surgery, report lower non-surgical site pain, and have lower chronic pain (Table 1B). We observed key differences between the MGI model’s training and test datasets: the training data had a higher proportion of females (53% vs 51%, *p* = 0.006) and more patients who experienced POU (21% vs 19%, *p* < 0.001) (Supplement 2). While we observed statistically significant differences between the training and test data for age, gender, Elixhauser Comorbidity Index, average pain, PROMIS physical score, chronic pain, hydrocodone/acetaminophen scripts, and tramadol scripts, these differences were small and not clinically meaningful.

**Table 1:** A MGI cohort characteristics by race. B MGI cohort characteristics by persistent opioid use.

A					
Characteristic	Overall^a^ *N* = 24,040	White^a^ *N* = 21,482	Black^a^ *N* = 1,175	Other^b^ *N* = 1,383	*p*-value^b^
Age, years	55 (42, 65)	56 (43, 66)	48 (36, 59)	48 (35, 61)	<0.001
Female	12,668 (53%)	11,239 (52%)	699 (59%)	730 (53%)	<0.001
Opioid-naive	11,508 (48%)	10,261 (48%)	487 (41%)	760 (55%)	<0.001
Elixhauser Comorbidity Index	3 (1, 5)	3 (1, 5)	3 (2, 6)	2 (1, 4)	<0.001
Average pain in week not at surgical site	2 (0, 4)	2 (0, 4)	3 (0, 6)	2 (0, 4)	<0.001
% Missing	**11**	**11**	**16**	**13**	
PROMIS physical 4-item score	18 (13, 20)	18 (13, 20)	17 (12, 20)	19 (14, 20)	0.002
% Missing	**14**	**14**	**20**	**17**	
Chronic pain	13,277 (55%)	11,846 (55%)	715 (61%)	716 (52%)	<0.001
% Missing	**<0.1**	**<0.1**	**0**	**0**	
Perceived Stress Scale	5 (2, 8)	5 (2, 8)	5 (2, 8)	5 (2, 8)	0.014
% Missing	**15**	**15**	**22**	**18**	
Life satisfaction	8 (5, 9)	8 (5, 9)	7 (5, 9)	8 (5, 9)	0.8
% Missing	**14**	**14**	**20**	**17**	
Hydrocodone/acetaminophen fills	0 (0, 1)	0 (0, 1)	0 (0, 1)	0 (0, 1)	<0.001
Tramadol fills	0 (0, 0)	0 (0, 0)	0 (0, 0)	0 (0, 0)	0.11
Persistent opioid use (outcome)	4879 (20%)	4348 (20%)	304 (26%)	227 (16%)	<0.001

B				
Characteristic	All^a^ *N* = 24,040	No POU^a^ *N* = 19,161	POU^a^ *N* = 4879	*p*-value^b^
Age, years	55 (42, 65)	55 (41, 66)	56 (45, 64)	<0.001
Female	12,668 (53%)	10,008 (52%)	2660 (55%)	0.004
Opioid-naive	11,508 (48%)	10,872 (57%)	636 (13%)	<0.001
Elixhauser Comorbidity Index	3 (1, 5)	3 (1, 4)	4 (2, 6)	<0.001
Average pain in week not at surgical site	2 (0, 4)	1 (0, 3)	4 (2, 6)	<0.001
% Missing	**11**	**11**	**12**	
PROMIS physical 4-item score	18.0 (13.0, 20.0)	19.0 (14.0, 20.0)	13.0 (9.0, 18.0)	<0.001
% Missing	**14**	**13**	**17**	
Chronic pain	13,277 (55%)	9370 (49%)	3907 (80%)	<0.001
% Missing	**<0.1**	**<0.1**	**0**	
Perceived Stress Scale	5.0 (2.0, 8.0)	4.0 (2.0, 7.0)	6.0 (3.0, 8.0)	<0.001
% Missing	**15**	**15**	**19**	
Life satisfaction	8.00 (5.00, 9.00)	8.00 (5.00, 9.00)	5.00 (4.00, 8.00)	<0.001
% Missing	**14**	**14**	**16**	
Hydrocodone/acetaminophen fills	0.00 (0.00, 1.00)	0.00 (0.00, 1.00)	1.00 (0.00, 7.00)	<0.001
Tramadol fills	0.00 (0.00, 0.00)	0.00 (0.00, 0.00)	0.00 (0.00, 1.00)	<0.001

^a^Statistics presented: median (IQR) for continuous variables; *n* (%) for categorical variables

^b^Statistical tests performed between White and Black patients (A) and between patients without and with persistent opioid use (B): chi-square test of independence for categorical variables, Wilcoxon rank-sum test for continuous variables

Predictor variables for the MGI model are listed in Supplement 1A. Variables used for matching are listed in Supplement 5.

For the refit claims model, the cohort comprised 11,508 opioid-naive patients. This cohort was predominantly non-Hispanic White (89%) and largely nonsmokers (58%) (Table 2A). The 12% of patients who experienced new POU had higher Charlson Comorbidity Index scores, greater preoperative benzodiazepine use, and comorbidities such as fibromyalgia and osteoarthritis (Table 2B). Compared to the test set, training set patients had lower Charlson Comorbidity Index scores and were less likely to have osteoarthritis (Supplement 3).

**Table 2:** A Refit claims cohort characteristics by race. B Refit claims cohort characteristics by new persistent opioid use.

A					
Characteristic	Overall^a^ *N* = 11,508	White^a^ *N* = 10,261	Black^a^ *N* = 487	Other^a^ *N* = 760	*p*-value^b^
Age, years	54 (40, 65)	55 (41, 66)	45 (32, 58)	46 (32, 59)	<0.001
Female	6034 (52%)	5346 (52%)	288 (59%)	400 (53%)	0.002
Smoking status					<0.001
Former	3674 (32%)	3381 (33%)	108 (22%)	185 (24%)	
Never	6690 (58%)	5860 (57%)	315 (65%)	515 (68%)	
Current	1090 (9.5%)	974 (9.5%)	61 (13%)	55 (7.2%)	
Unknown	54 (0.5%)	46 (0.4%)	3 (0.6%)	5 (0.7%)	
Education					
Less than a high school degree	0.06 (0.03, 0.09)	0.06 (0.03, 0.09)	0.09 (0.05, 0.13)	0.05 (0.03, 0.09)	<0.001
High school degree and/or some college	0.60 (0.43, 0.71)	0.61 (0.44, 0.71)	0.61 (0.47, 0.69)	0.48 (0.29, 0.66)	0.7
Bachelor’s degree or higher	0.32 (0.20, 0.52)	0.32 (0.20, 0.52)	0.28 (0.16, 0.45)	0.44 (0.23, 0.68)	<0.001
% Missing	**8.4**	**8.3**	**11**	**8.4**	
Charlson Comorbidity Index	2 (1, 6)	3 (1, 6)	1 (0, 4)	1.0 (0.0, 4.0)	<0.001
Drugs					
Preoperative benzo use	15	15	11	11	0.007
Lamotrigine fills	0 (0, 0)	0 (0, 0)	0 (0, 0)	0 (0, 0)	0.3
Diagnoses					
Abdominal pain	2223 (19%)	1949 (19%)	114 (23%)	160 (21%)	0.016
Arthritis joint pain	5345 (46%)	4732 (46%)	242 (50%)	371 (49%)	0.12
Back pain	1051 (9.1%)	918 (8.9%)	63 (13%)	70 (9.2%)	0.003
Fibromyalgia	456 (4.0%)	403 (3.9%)	27 (5.5%)	26 (3.4%)	0.075
Headache (incl. migraine)	2001 (17%)	1764 (17%)	106 (22%)	131 (17%)	0.009
Headache (excl. migraine)	1339 (12%)	1173 (11%)	79 (16%)	87 (11%)	0.001
Osteoarthritis	2423 (21%)	2206 (21%)	101 (21%)	116 (15%)	0.7
Non-traumatic joint pain	3849 (33%)	3386 (33%)	179 (37%)	284 (37%)	0.085
Surgery					0.5
Major	10,769 (97%)	9590 (97%)	348 (96%)	724 (97%)	
% Missing	**3.9**	**4.0**	**3.5**	**1.8**	
New persistent opioid use (outcome)	1428 (12%)	1298 (13%)	62 (13%)	68 (8.9%)	>0.9

B				
Characteristic	Overall^a^ *N* = 11,508	No New POU^a^ *N* = 10,080	New POU^a^ *N* = 1428	*p*-value^b^
Age, years	54 (40, 65)	54 (39, 65)	57 (45, 67)	<0.001
Female	6034 (52%)	5254 (52%)	780 (55%)	0.077
Smoking status				<0.001
Former	3674 (32%)	3122 (31%)	552 (39%)	
Never	6690 (58%)	5976 (59%)	714 (50%)	
Current	1090 (9.5%)	936 (9.3%)	154 (11%)	
Unknown	54 (0.5%)	46 (0.5%)	8 (0.6%)	
Education				
Less than a high school degree	0.06 (0.03, 0.09)	0.06 (0.03, 0.09)	0.06 (0.04, 0.10)	<0.001
High school degree and/or some college	0.60 (0.43, 0.71)	0.60 (0.43, 0.71)	0.63 (0.48, 0.72)	<0.001
Bachelor’s degree or higher	0.32 (0.20, 0.52)	0.33 (0.20, 0.53)	0.30 (0.18, 0.47)	<0.001
% Missing	**8.4**	**8.6**	**7.4**	
Charlson Comorbidity Index	2.0 (1.0, 6.0)	2.0 (1.0, 5.0)	4.0 (1.0, 8.0)	<0.001
Drugs				
Preoperative benzo use	1,712 (15%)	1,411 (14%)	301 (21%)	<0.001
Lamotrigine fills	0 (0, 0)	0 (0, 0)	0 (0, 0)	0.4
Diagnoses				
Abdominal pain	2223 (19%)	1919 (19%)	304 (21%)	0.044
Arthritis joint pain	5345 (46%)	4672 (46%)	673 (47%)	0.6
Back pain	1051 (9.1%)	893 (8.9%)	158 (11%)	0.007
Fibromyalgia	456 (4.0%)	375 (3.7%)	81 (5.7%)	<0.001
Headache (incl. migraine)	2001 (17%)	1740 (17%)	261 (18%)	0.3
Headache (excl. migraine)	1339 (12%)	1177 (12%)	162 (11%)	0.7
Osteoarthritis	2423 (21%)	2061 (20%)	362 (25%)	<0.001
Non-traumatic joint pain	3849 (33%)	3364 (33%)	485 (34%)	0.7
Surgery				<0.001
Major	10,769 (97%)	9522 (98%)	1247 (95%)	
% Missing	**3.9**	**3.2**	**8.5**	

^a^Statistics presented: median (IQR) for continuous variables; *n* (%) for categorical variables; education variables are census-tract proportions, presented as median (IQR).

^b^Statistical tests performed between White and Black patients (A) and between patients without and with new persistent opioid use (B): chi-square test of independence for categorical variables, Wilcoxon rank-sum test for continuous variables. Note that predictor variables for the refit claims model are listed in Supplement 1B. Variables used for matching are listed in Supplement 5.

### Robustness of performance differences in racial subgroups

#### MGI model

**Fig. 1:**
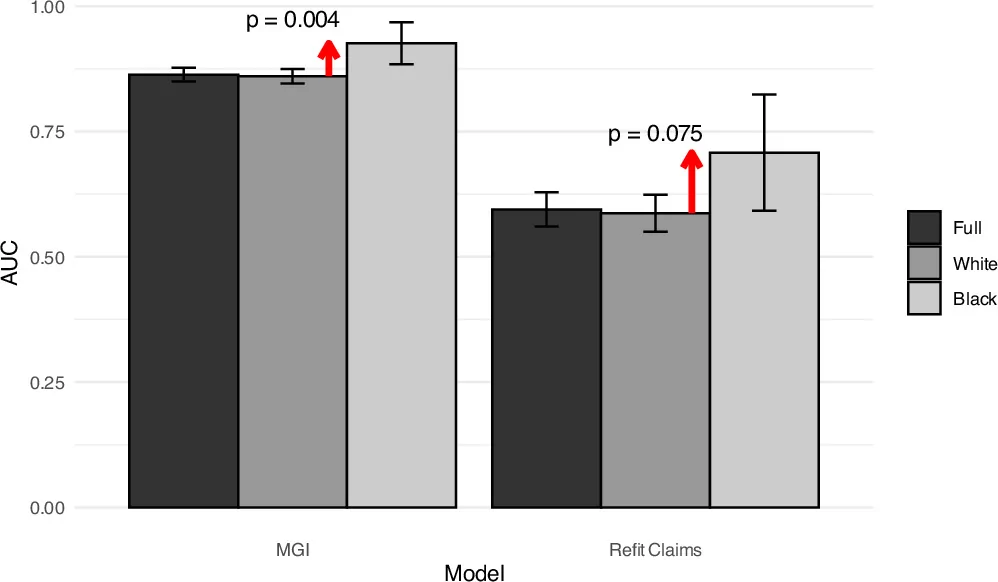
Comparison of model performance (AUC) for Overall, White, and Black patients. Error bars capture the 95% CI of the AUC. *p*-values are for difference in AUC between White and Black patients based on the DeLong test

As previously reported^21^, the MGI model had an AUC of 0.86 (0.85–0.88) in predicting POU overall, with an AUC of 0.86 (0.85–0.88) in White patients and 0.93 (0.88–0.97) in Black patients (Fig. 1). The MGI model performed better in Black patients with test AUC 0.066 higher than in the White population (*p* = 0.004).

#### Refit claims model

In the opioid-naive subpopulation, the refit claims model had an AUC of 0.59 (0.56–0.63) overall, 0.59 (0.55–0.62) in White patients, and 0.71 (0.59–0.82) in Black patients (Fig. 1). While the refit claims model had a higher point estimate in Black patients with a testing AUC 0.121 higher than in the White population, this finding was not statistically significant (*p* = 0.075), potentially attributable to the reduced sample size of opioid-naive patients (testing data *N* = 2749).

### Baseline characteristics in Black and White patients

**Fig. 2:**
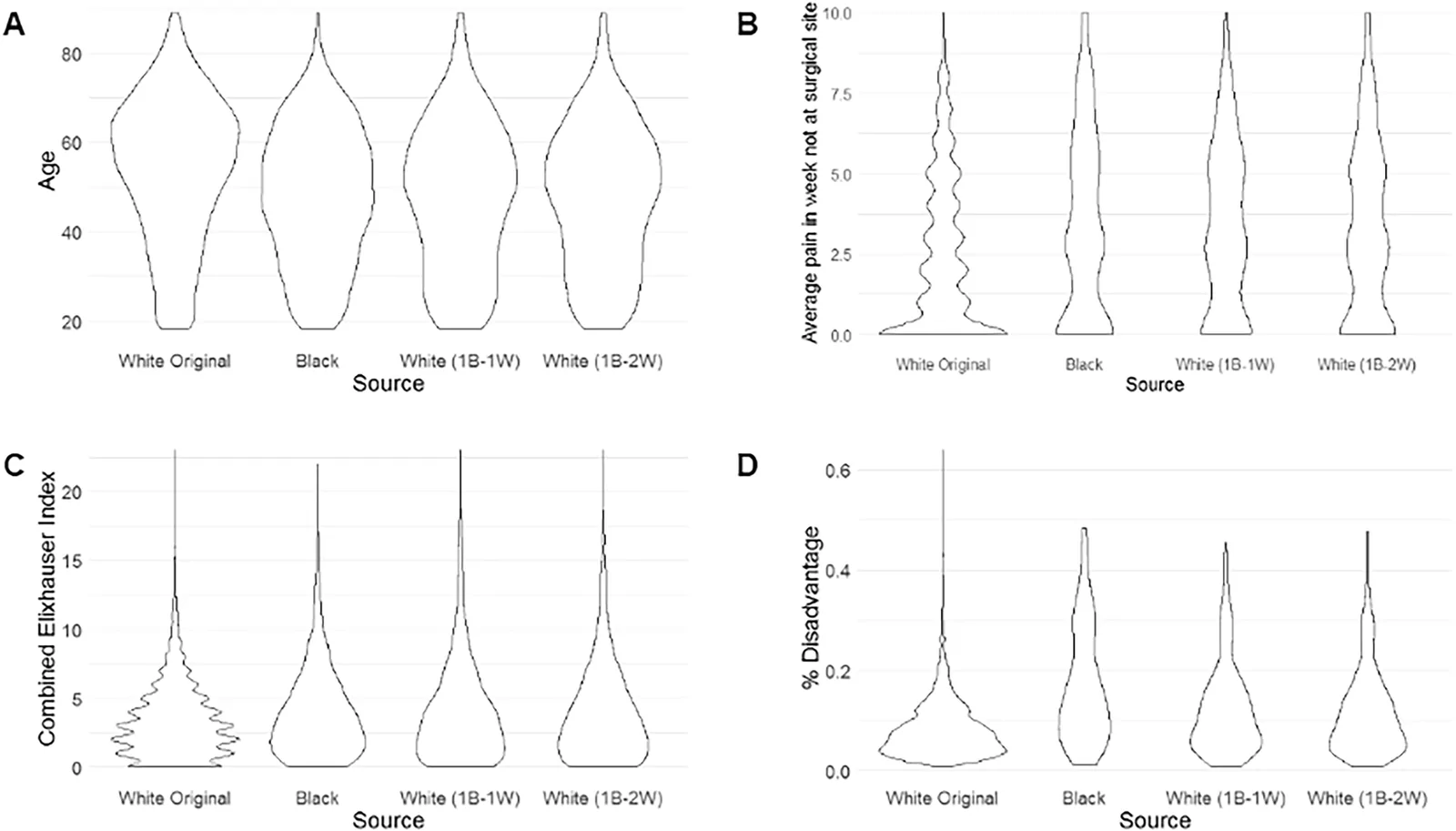
Distributions of baseline patient characteristics in the Michigan Genomics Initiative (MGI) cohort by race and matching status. (**A**) Age in years. (**B**) Average pain in week not at surgical site (0 to 10 scale). (**C**) Combined Elixhauser Comorbidity Index. (**D**) Census tract-level neighborhood disadvantage from the National Neighborhood Data Archive (NaNDA), scored from 0 to 1. Each violin shows the distribution of the variable in one group, with width proportional to the density of patients at each value. White Original refers to unmatched White patients; Black refers to Black patients; White (1B-1W) and White (1B-2W) refer to White patients matched to Black patients at 1 Black:1 White and 1 Black:2 White, respectively.

To identify factors that may be contributing to the performance differences observed above, we compared baseline characteristics between Black and White patients. Figure 2 displays covariate distributions for the original White cohort, Black patients, and the matched White cohorts (1B-1W and 1B-2W; the matching procedure is described below). In the overall MGI cohort, Black patients were younger than the original White cohort (Fig. 2A). On the measure of average pain, Black patients had a wider distribution of reported pain compared to that for White patients (Fig. 2B). On the Elixhauser Comorbidity Index, White patients had a higher skew, compared to the relatively smoother distribution for Black patients (Fig. 2C). NaNDA (National Neighborhood Data Archive)-defined neighborhood disadvantage levels were more homogenous among White patients, and the overall neighborhood disadvantage score was lower in White patients as compared to Black patients (Fig. 2D, Supplement 4).

### Subgroup matching

**Fig. 3:**
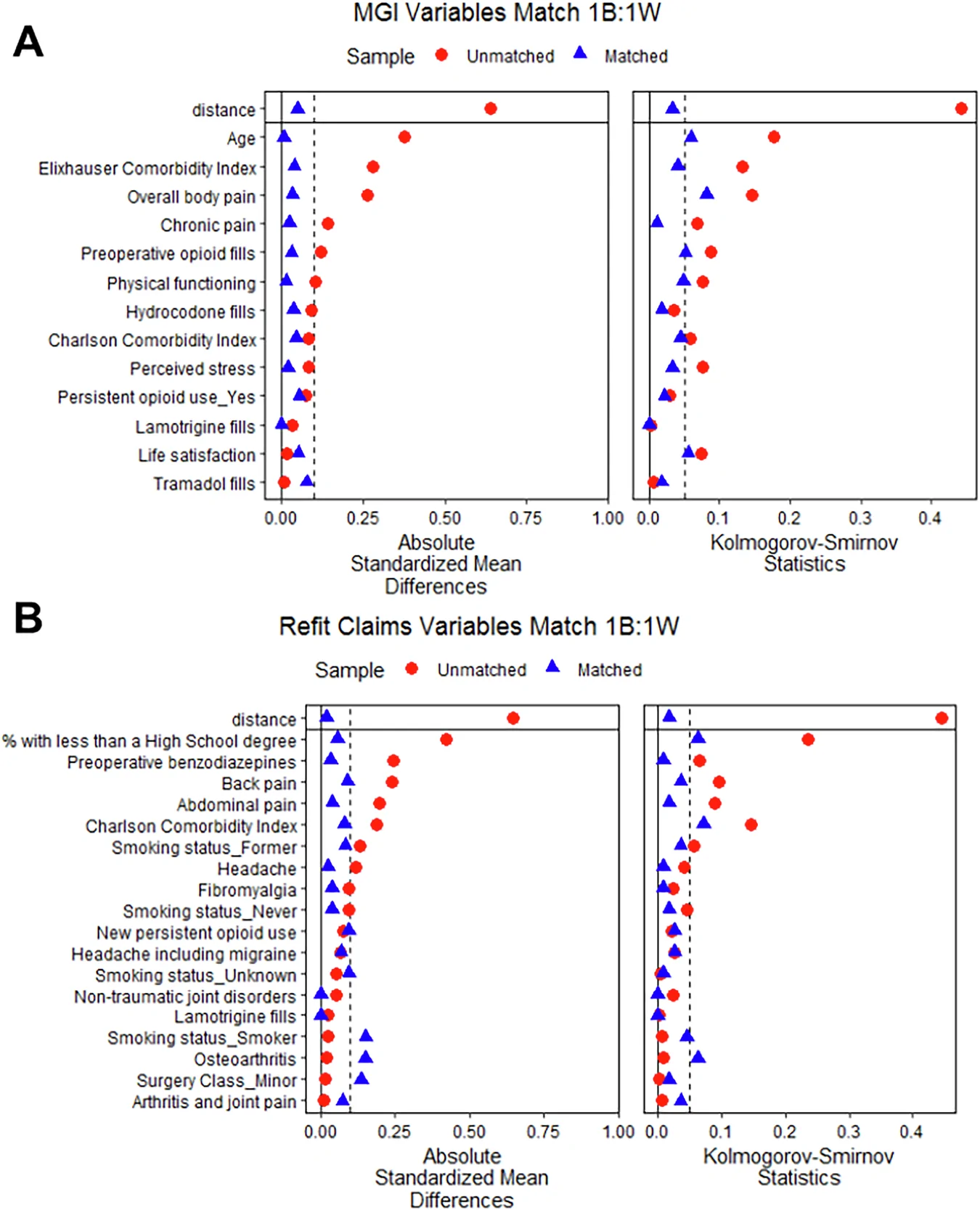
Love plots of matched cohort ratio 1 Black:1 White. **A** Depicts the absolute standardized mean differences and the KS statistics for select covariates in the MGI model between Black and White patients in the data before (red circles) and after matching (blue triangles). **B** Depicts the absolute standardized mean differences and the KS statistics for select covariates in the Refit Claims model between Black and White patients in the data before (red circles) and after matching (blue triangles).

If subgroup model performance differences stemmed from observable baseline characteristics, matching patients across subgroups could potentially attenuate these differences, suggestive of upstream bias. We used subgroup matching to characterize the role of patient characteristics in contributing to model performance differences. Each Black patient was matched to their nearest one or two White patients without duplication (1B-1W and 1B-2W matching) based on the consolidated set of variables used in the MGI and refit claims variables, excluding the outcome variables (Supplement 5). Covariate balance between White and Black patients improved after matching in the 1B-1W cohort, with matched White patients more closely resembling Black patients than unmatched White patients did (Fig. 3 and Supplement 6, 7). Improved covariate balance after matching was similarly observed in the 1B-2W cohort (Supplement 8).

#### Test-only matching: train on full training data, evaluate on matched testing data

**Fig. 4:**
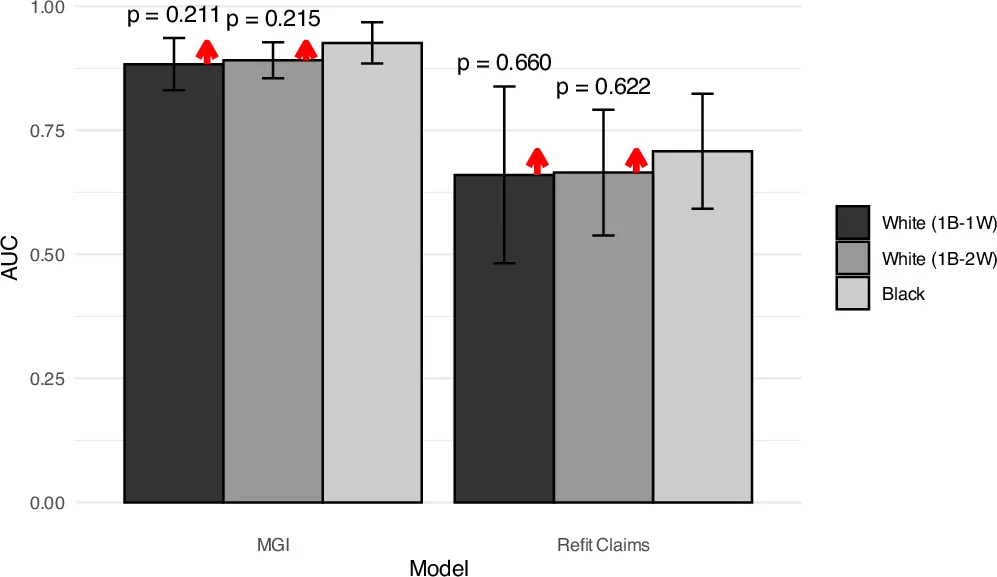
Comparison of model performance (AUC) for Matched White (1B-1W), Matched White (1B-2W) and Black patients. Error bars capture the 95% CI of the AUC. *p*-values are for differences in AUC between White and Black patients based on the DeLong test.

The MGI model performance on matched White patients increased from an original AUC of 0.86 to a matched AUC of 0.88 (0.83–0.94) and 0.89 (0.86–0.93) in the 1B-1W and 1B-2W matched ratio test cohorts, respectively (Fig. 4). The matched AUC in Black patients was similar at 0.926 (0.88–0.97). The differences between Black and White patients were no longer statistically significant for both the (1B-1W) and (1B-2W) cohorts. A similar finding was observed for the refit claims model.

**Fig. 5:**
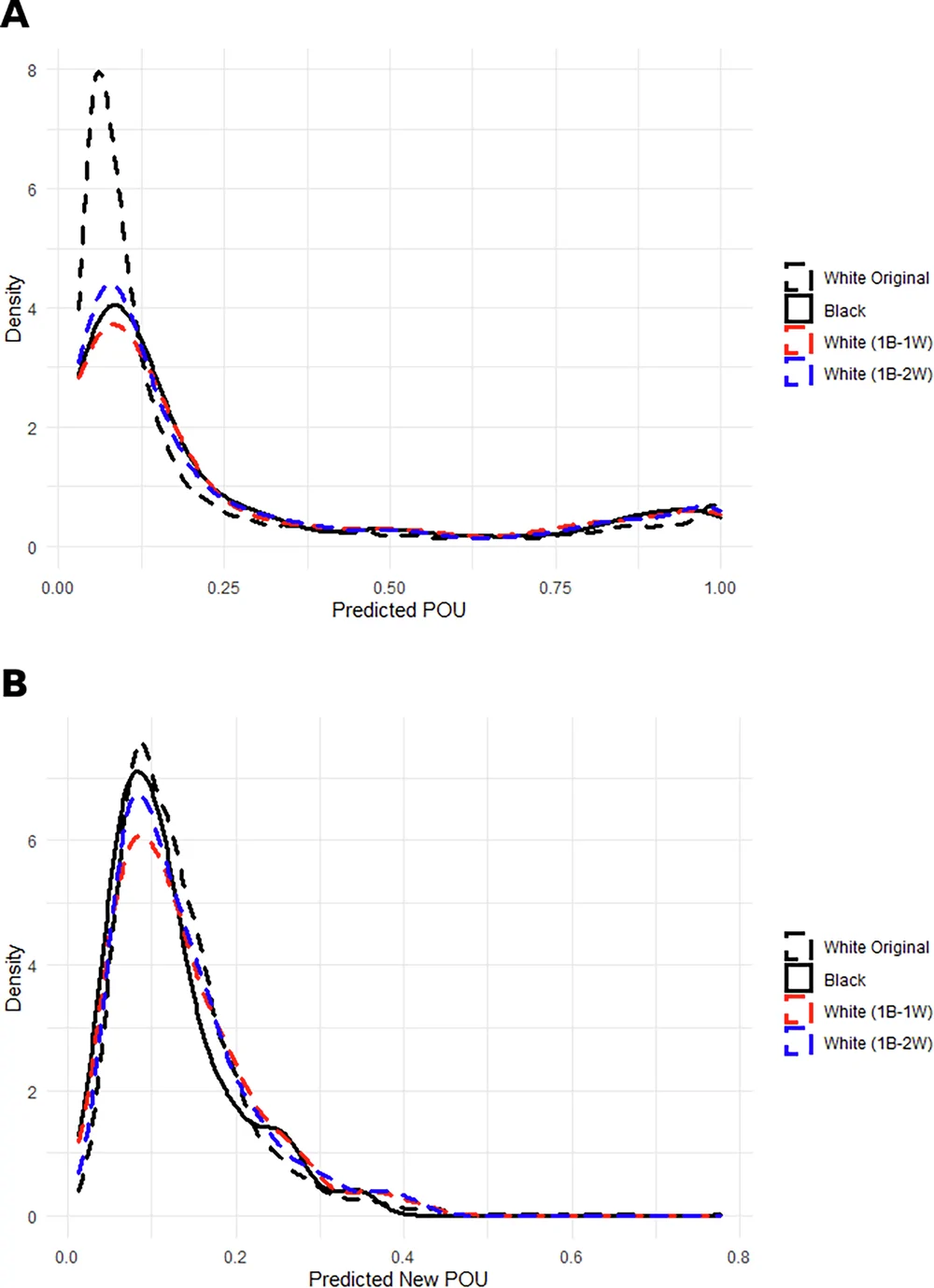
Density distributions of predicted probabilities of persistent opioid use in Black, White, and matched White patients. (**A**) Michigan Genomics Initiative (MGI) model: predicted probability of persistent opioid use (POU) after surgery. (**B**) Refi t claims model: predicted probability of new POU after surgery among opioid-naive patients. In both panels, each curve shows the density of predicted probabilities for one group: White Original refers to unmatched White patients (black dashed line); Black refers to Black patients (black solid line); White (1B-1W) refers to White patients matched to Black patients at 1 Black:1 White (red dashed line); and White (1B-2W) refers to White patients matched at 1 Black:2 White (blue dashed line).

For the MGI model, the density distribution of predicted probabilities of the outcome is more similar between Black patients and the matched cohorts compared to that of the original White patient cohort in the MGI model (Fig. 5A). A similar convergence was observed for the refit claims model (Fig. 5B). Given that the outcome was not included in the matching step, this suggests a matching success not just in terms of the patient observables, but also their behavior patterns of interest.

#### Full matching with training: trained on matched training data, evaluate on matched testing data

Reduced performance differences between the White and Black patients in the matched cohorts were also seen when models were re-trained using matched training data (Supplement 9).

#### Other performance metrics

To characterize model fairness more broadly than AUC alone allows, we examined a broader set of performance metrics across subgroups. Supplement 10 presents performance metrics for the MGI model across White patients, matched White patients, and Black patients, including sensitivity, specificity, positive predictive value (PPV), negative predictive value (NPV), Matthews Correlation Coefficient (MCC), and proportion of positives. Five of the six additional metrics showed a pattern consistent with the AUC findings, with more favorable performance in Black patients than unmatched White patients that attenuated after matching. Specificity was the exception, being lower in Black patients, possibly reflecting their higher outcome prevalence (26% vs 20%), which shifts predicted scores upward and produces more false positives at a fixed threshold. After subgroup matching, metrics in the matched White cohorts were more closely aligned with those in the Black cohort, supporting the role of observed characteristics in contributing to model performance differences.

## Discussion

In this retrospective study using the longitudinal MGI cohort, we found that two opioid prediction models exhibited a higher point estimate for AUC among Black patients as compared to White patients, a finding that was statistically significant for one of the two models. A comparison of baseline patient characteristics revealed greater heterogeneity among Black patients in terms of predicted risk, comorbidities, and socioeconomic disadvantage measures. Matched subgroups of White patients in the testing data had a higher AUC as compared to the full cohort of White patients, with an AUC similar to that of Black patients. The matched White cohort had a more similar distribution of predicted outcome probabilities to Black patients as compared to the full cohort of White patients. This pattern held whether models were trained on the full training data or the matched training data. Attenuation of differences between Black patients and White patients following matching was also observed among the other performance metrics.

Our findings suggest that differences in baseline patient characteristics between Black and White patients are sufficient to explain much of the observed AUC disparity, though we cannot fully disentangle the contributions of the data distribution from those of the model itself. Because AUC is a measure of discriminative performance, the presence of extremely high- and low-risk patients in a dataset can inflate the AUC, as was seen in Black patients in our study. Conversely, in groups with more uniformly low- or average-risk patients (i.e., a more homogenous risk population), the model cannot readily differentiate events from non-events, resulting in a lower AUC. This phenomenon has been recognized in the criminal justice system^25^, where higher AUCs have been observed in female subgroups due to greater variation in types of crimes and sentencing. In our study, Black patients were more heterogeneous with respect to both their baseline characteristics and distribution of model probabilities.

Heterogeneity is not inherently problematic, as long as the heterogeneous characteristics for a population are not *also* important predictors of risk. In our study, heterogeneous baseline characteristics *were* linked to the patients’ risk. White patients matched on baseline characteristics exhibited more similar risk profiles and observed AUCs to Black patients. Thus, belonging to a uniformly lower-risk group (lower AUC and lower persistent opioid use) in our study would be preferable to being in a varying-risk group (higher AUC). When AUCs for two subgroups become similar after matching, upstream biases related to the representativeness of patient subgroups should be considered. On the other hand, if a model’s performance does not change in response to matching, this suggests the gap may not be fully explained by the observed covariates alone. For example, a recent study comparing sex differences in acute kidney injury models found that matching did not reduce subgroup differences^26^. Unmeasured confounders, such as income or insurance type, may reflect unequal access to care that matching on available variables cannot fully capture. This could be evaluated by comparing patients who received care to the broader eligible population to identify factors linked to differential access. Predictors may also serve as proxies for race in ways that do not generalize across groups, or outcome labels may be measured differentially due to variation in clinical documentation, scrutiny, or missingness. Where the source of a persistent gap is better understood, interventions such as targeted matching on relevant access variables, fairness constraints, feature auditing, or subgroup-specific models may be appropriate, though each involves tradeoffs with overall performance.

Our findings have major implications for the evaluation of fairness in predictive AI models. In May of 2024, the US Department of Health and Human Services Office for Civil Rights published a final rule—one that remains in effect—requiring that organizations using AI “must make reasonable efforts to mitigate the risk of discrimination.”^27^ This has been followed by state legislation aiming to promote fairness in AI^28^. Measuring subgroup performance is a common aspect of such efforts. The “AUC gap,” or the largest difference between subgroups for AUC, has been proposed as a way of checking if a model is fair^29^, with the implication that lower AUCs are worse. On the contrary, we demonstrate that lower AUCs may be driven in part by homogeneity of population risk profiles and may even be preferable. If AUC is to be used to compare population subgroups, we recommend that any stratified reporting of AUC across subgroups should be accompanied by a matched analysis.

Our study has limitations. First, the matching approach may fail if there are insufficient patients in the majority group to match with the minority group, and because training a model generally requires a larger sample size than validation, 1:1 matching on the training dataset may result in a sample too small for model training^30^. Second, while our matching framework provides evidence that covariate differences contribute to observed AUC disparities, it cannot rule out alternative explanations such as unmeasured confounding, label noise, or differential measurement error; our findings are therefore best interpreted as consistent with upstream influences rather than as definitive proof of upstream bias. Third, our primary indicator of structural disadvantage, neighborhood-level socioeconomic disadvantage (NaNDA), is a census tract-level proxy; individual-level measures of care access, diagnostic coding quality, and label reliability were not available, and the coarse spatial resolution of these measures limits the specificity of upstream inference. Fourth, this retrospective study was conducted using data from a large academic medical center, potentially limiting its generalizability. Finally, AUC differences within subgroups represent a small portion of a holistic evaluation of model fairness.

Despite these limitations, our study has important implications for assessing model fairness and bias. Our findings challenge the notion that a high AUC in a demographic minority is inherently reassuring regarding model performance. Instead, a higher AUC may reflect greater upstream bias and increased risk for adverse outcomes among minorities, an issue that can be investigated using our matching framework. Our study underscores the importance of measuring and accounting for demographic characteristics to ensure fairness across subgroups.

## Methods

### Study cohort

We used data from the Michigan Genomics Initiative (MGI), a longitudinal biorepository at the University of Michigan started in 2012^21,31,32^. The repository enrolls perioperative patients, integrates electronic health records (EHRs) with self-reported questionnaire data, and links to Michigan’s Prescription Drug Monitoring Program (PDMP) via a data-sharing agreement, enabling high-quality tracking of opioid fills before and after surgery^33^. Our cohort included pre-surgical patients enrolled in the MGI cohort who underwent index surgery within 90 days of enrollment between January 1, 2015, and July 3, 2018. We excluded patients living outside Michigan as PDMP data were limited to the state of Michigan. This study was approved by the Institutional Review Board at Michigan Medicine (Ann Arbor, MI; HUM00149878), and the need for consent was waived by the same board. All methods were carried out in accordance with relevant guidelines and regulations governing human subjects research.

### Data source

Our primary data source for this study is MGI data^33^. It is supplemented with census tract and zip code-level education variables (e.g., proportion with less than a high school diploma) from the National Neighborhood Data Archive (NaNDA), a publicly available resource comprising physical, economic, demographic, and social environmental variables at various spatial scales^34^.

### Statistical analyses

We evaluated two published persistent opioid use prediction models using this data: one originally trained using MGI data^21^ by Singh et al., and another that was originally trained on claims data^24^ by Hur et al.

#### MGI model

Since the Singh et al. model was originally developed using the MGI cohort, we refer to it as the *MGI model* and used the original published coefficients.

#### Refit claims model

The original model in Hur et al. was developed with opioid-naive adults aged 18–64 using Optum’s Clinformatics Data Mart, which contains insurance claims data. Our EHR-based MGI data lacked some variables that were present in the Hur et al. prediction model. Specifically, the latter used individual patient education levels which were extensively missing and inconsistent in the EHR data. We replaced this with the NaNDA education attainment distribution from the patient’s census tract (eg: proportions with or without a high school diploma, and with a Bachelor’s degree or higher)^34^. Although the Hur et al. model was originally trained using claims data, we refit it using MGI data for the purposes of this study due to differences in the original claims data and our MGI EHR-based data that prevented our using it in its original form. We refer to the Hur et al. model, trained with these variables as the *refit claims model*.

Additionally, since the Hur et al. model applies only to opioid-naive patients, we restricted training and evaluation of the refit claims model to an opioid-naive cohort, defined as patients who had not filled an opioid prescription within 365 to 31 days prior to their index surgery. Results from our *MGI* and *refit claims* models are not directly comparable, but test the robustness of our findings. Because the refit claims model is applied to a population that differs substantially from the original Hur et al. development cohort in demographics, comorbidity profiles, and outcome definition, some attenuation of absolute performance is expected, as is common when prediction models are transported across populations^35^.

### Outcomes

The two models we evaluated were trained to predict different outcomes. The MGI model predicts persistent opioid use (POU) after surgery, whereas the refit claims model predicts *new* POU after surgery among opioid-naive patients. POU after surgery was defined similarly to Howard et al.^36^, identified if a patient filled: (1) an opioid prescription twice during the perioperative period (30 days before surgical admission to 3 days after discharge) and/or (2) an opioid prescription during post-discharge days 4–90, and (3) an opioid prescription during post-discharge days 91–180. New POU was defined as having filled an opioid prescription during post-discharge days 91–180.

#### MGI model

The logistic regression model to predict POU included key demographic, clinical, and patient-reported predictors and is detailed in Supplement 1A. This model was originally developed using the same data source as in this study.

#### Refit claims model

The XGBoost model predicting new POU included demographic and clinical predictors, detailed in Supplement 1B.

#### Model training and testing cohorts

Data were split by index surgery date, with surgeries before July 5th, 2017 assigned to the training cohort and those after to the testing cohort. The MGI model used the same coefficients as the original manuscript^21^. The refit claims model was refit (i.e., trained) using the training cohort, tuned using 5-fold cross-validation with a maximum of 5000 iterations, and then evaluated in the testing cohort. The final model parameters were: eta = 0.3, max depth = 1, lambda = 5. We conducted subgroup analyses for all models to evaluate model performance separately in Black and White subgroups.

#### Differences between subgroups

We compared the following observed characteristics between Black and White patient cohorts: age, average pain in week not at surgical site, the Elixhauser Comorbidity Index, and socioeconomic status (SES), and NaNDA’s neighborhood *disadvantage* variable (ranging from 0 to 1). The *disadvantage* variable averages four census indicators: proportion of female-headed families with children, proportion of households receiving public assistance income or food stamps; proportion of families below the federal poverty level; proportion of population aged 16+ unemployed. The MGI model does not include SES-related predictors, while the refit claims model uses education level as a predictor.

#### Subgroup matching

We applied two matching-based approaches to characterize the role of patient characteristics in contributing to model performance differences:

1. *Test-only Matching (primary analysis)*: applying the original models (trained on the full, unmatched training data) to the matched testing cohorts, and
2. *Full Matching with Training (secondary analysis)*: re-training models on matched training data and evaluating them on the matched testing cohorts.

The first approach evaluates upstream bias within the testing set alone, whereas the second approach evaluates upstream bias within the training and testing sets. While the second approach allows for a more comprehensive evaluation of upstream bias, it is limited by the need to re-train models and a reduction in sample size in the training data due to matching.

In the first approach, models trained on the full unmatched training data were evaluated on a subset of the testing data in which White patients were matched to Black patients based on observed characteristics. The MatchIt^37^ package was used to apply a case-control propensity score matching approach with a logistic regression generalized linear model. Each Black patient was matched to the nearest White patient by propensity score, without replacement, using Black:White ratios of 1:1 and 1:2. Matching included all predictor variables from both the MGI and refit claims models, along with gender. For education, we used only the percentage of the census tract population with less than a high school degree to avoid collinearity among the education variables. Match quality was assessed using “love plots” to compare matched and unmatched cohort characteristics^38^. In the second approach, the models were retrained on matched training data and similarly evaluated on matched testing cohorts. AUC by subgroup was compared using the DeLong test^39^.

#### Other performance metrics

In addition to subgroup AUC comparisons, we evaluated a broader set of performance metrics across subgroups: Sensitivity, Specificity, PPV, NPV, the MCC, and proportion of positives. We calculated these metrics for Black patients, unmatched White patients, and matched White patients. This evaluation allowed us to characterize model fairness more broadly.

#### Missing data

For socioeconomic level variables such as education, we imputed missing values using the mean of other patients within the same three-digit zip code. Remaining patient-level missing values were imputed using bootstrapped aggregated (bagged) trees. For missing data on lamotrigine fill quantities, however, we imputed values for patients with a prescription but unknown quantities using the median of patients with known non-zero lamotrigine quantities.

## Supplementary information

Supplement_July2026

## Data Availability

We used data from the Michigan Genomics Initiative (MGI), a longitudinal biorepository at the University of Michigan started in 2012. Patient confidentiality prevents the sharing of these data publicly. However, the data underlying the study’s results are available from the Michigan Genomics Initiative (https://sites.lsa.umich.edu/aidhi-data-details/genetic-data-2/) for researchers who meet the criteria for confidential data access. Researchers may request access via the form on the MGI website (https://aidhi.umich.edu/mgi-community/#mgi-data), subject to eligibility and approval.

## Code availability

The R code used to conduct the analyses in this study is available from the corresponding author upon reasonable request.
