# Supplementary material for: Paradoxical inflation of prediction model performance measures in minority patient subgroups: the role of upstream bias
Source: Npj Health Syst. 2026 Oct 21;3:99. doi: 10.1038/s44401-026-00158-0 (PMC13687106; doi:10.1038/s44401-026-00158-0)
Supplement: Supplementary file 1 [file 44401_2026_158_MOESM1_ESM.pdf]

Supplement

Predictor Category	Variable
**Demographic**	Age
**Clinical/Patient-Reported**	Elixhauser Comorbidity Index
	Overall Preoperative Opioid Fill Counts from PDMP Data
	Specific counts of hydrocodone-acetaminophen
	Specific counts of tramadol
	Patient-Reported Outcomes Measurement Information System (PROMIS) physical 4-item score
	Average Pain in a week at the surgical site
	Average Pain in a week not at the surgical site
	Chronic Pain
	Perceived Stress

Supplement 1A: Predictor Variables - MGI Model

Predictor Category	Variable
**Demographic**	Age
	Education Level
**Clinical**	History of Depression
	History of Anxiety
	Abdominal Pain
	Back Pain
	Joint Disorders
	Arthritis and Joint Pain
	Osteoarthritis
	Headache including Migraine
	Fibromyalgia
	Number of Lamotrigine prescriptions within 1 year prior to the surgery
	Preoperative Benzodiazepine Exposure
	Tobacco Use
	Type of surgical procedure (major/minor) from ICD-9 procedure codes

Supplement 1B: Predictor Variables - Refit Claims Model

Source	Overall Train[1] N = 18,239	Overall Test[1] N = 5,801	Overall p-val[2]	White Train[1] N = 16,301	White Test[1] N = 5,181	White p-val[2]	Black Train[1] N = 906	Black Test[1] N = 269	Black p-val[2]
**Age, years**	55 (41, 65)	57 (44, 66)	<0.001	55 (42, 65)	58 (45, 67)	<0.001	48 (35, 59)	50 (40, 61)	0.039
**Female**	9,702 (53%)	2,966 (51%)	0.006	8,603 (53%)	2,636 (51%)	0.017	559 (62%)	140 (52%)	0.005
**Opioid-naive**	8,759 (48%)	2,749 (47%)	0.4	7,807 (48%)	2,454 (47%)	0.5	376 (42%)	111 (41%)	>0.9
**Elixhauser Comorbidity Index**	3 (1, 5)	3 (1, 5)	<0.001	3 (1, 5)	3 (1, 5)	<0.001	3 (1, 5)	4 (2, 6)	<0.001
**Average pain in week not at surgical site**	2 (0, 4)	2 (0, 4)	0.011	2 (0, 4)	2 (0, 4)	0.014	3 (0, 6)	3 (0, 6)	0.8
% Missing	11	12		11	12		16	16	
**PROMIS physical 4-item score**	18 (13, 20)	18 (12, 20)	<0.001	18 (13, 20)	18 (12, 20)	<0.001	17 (12, 20)	17 (12, 20)	0.4
% Missing	14	15		13	14		19	24	
**Chronic pain**	9,976 (55%)	3,301 (57%)	0.003	8,902 (55%)	2,944 (57%)	0.005	544 (60%)	171 (64%)	0.3
% Missing	0	<0.1		0	<0.1		0	0	
**Perceived stress scale**	5 (2, 8)	5 (2, 8)	0.9	5 (2, 8)	5 (2, 8)	0.7	5 (2, 8)	5 (2, 8)	0.8
% Missing	15	16		15	16		21	25	
**Life satisfaction**	8 (5, 9)	7 (5, 9)	0.2	8 (5, 9)	7 (5, 9)	0.095	7 (5, 9)	8 (5, 9)	>0.9
% Missing	14	15		14	14		19	25	
**Hydrocodone/ acetaminophen # prescriptions**	0 (0, 1)	0 (0, 1)	0.014	0 (0, 1)	0 (0, 1)	0.023	0 (0, 1)	0 (0, 1)	0.07
**Tramadol # prescriptions**	0 (0, 0)	0 (0, 0)	0.011	0 (0, 0)	0 (0, 0)	0.009	0 (0, 0)	0 (0, 0)	0.9
**Persistent opioid use (outcome)**	3,799 (21%)	1,080 (19%)	<0.001	3,385 (21%)	963 (19%)	<0.001	246 (27%)	58 (22%)	0.066

Supplement 2: MGI Cohort Characteristics Train Test Split

1 Statistics presented: median (IQR) for continuous variables; n (%) for categorical variables
2 Statistical tests performed: Wilcoxon rank-sum test; chi-square test of independence

Characteristic	Overall Train[1] N = 8,759	Overall Test[1] N = 2,749	Overall p-val[2]	White Train[1] N = 7,807	White Test[1] N = 2,454	White p-val[2]	Black Train[1] N = 376	Black Test[1] N = 111	Black p-val[2]
**Age, years**	54 (40, 65)	56 (41, 66)	<0.001	55 (41, 65)	57 (42, 67)	<0.001	44 (32, 57)	48 (36, 60)	0.1
**Female**	4,638 (53%)	1,396 (51%)	0.047	4,117 (53%)	1,229 (50%)	0.22	2224 (60%)	64 (58%)	0.7
**Smoking status**			0.4			0.4			0.5
**Former**	2,820 (32%)	854 (31%)		2,600 (33%)	781 (32%)		79 (21%)	29 (26%)	
**Never**	5,054 (58%)	1,636 (60%)		4,423 (57%)	1,437 (59%)		245 (65%)	70 (63%)	
**Smoker**	842 (9.6%)	248 (9.0%)		748 (9.6%)	226 (9.2%)		50 (13%)	11 (9.9%)	
**Unknown**	43 (0.5%)	11 (0.4%)		36 (0.5%)	10 (0.4%)		2 (0.5%)	1 (0.9%)	
**Education**									
**Less than a high school degree**	0.06 (0.03, 0.09)	0.06 (0.03, 0.10)	0.006	0.06 (0.03, 0.09)	0.06 (0.03, 0.10)	0.1	0.09 (0.05, 0.13)	0.09 (0.05, 0.14)	>0.9
**High school degree and/or some college**	0.60 (0.43, 0.71)	0.61 (0.44, 0.71)	0.017	0.61 (0.43, 0.71)	0.62 (0.44, 0.71)	0.064	0.61 (0.47, 0.69)	0.62 (0.49, 0.70)	0.7
**Bachelor's degree or higher**	0.33 (0.20, 0.53)	0.32 (0.19, 0.52)	0.004	0.32 (0.20, 0.52)	0.31 (0.19, 0.51)	0.022	0.28 (0.17, 0.45)	0.26 (0.15, 0.45)	0.5
**% Missing**	**8.3**	**8.8**		**8.1**	**8.7**		**13**	**8.1**	
**Charlson Comorbidity Index**	2 (1, 5)	3 (1, 6)	<0.001	3 (1, 6)	3 (1, 7)	<0.001	1 (0, 4)	2 (0, 5)	0.2
**Drugs**									
**Preoperative benzo use**	1,311 (15%)	401 (15%)	0.6	1,215 (16%)	361 (15%)	0.3	44 (12%)	9 (8.1%)	0.3
**Lamotrigine fills**	0 (0, 0)	0 (0, 0)	0.6	0 (0, 0)	0 (0, 0)	0.5	0 (0, 0)	0 (0, 0)	>0.9
**Diagnoses**									
**Abdominal pain**	1,643 (19%)	580 (21%)	0.007	1,441 (18%)	508 (21%)	0.013	81 (22%)	33 (30%)	0.073
**Arthritis joint pain**	4,108 (47%)	1,237 (45%)	0.081	3,618 (46%)	1,114 (45%)	0.4	191 (51%)	51 (46%)	0.4
**Back pain**	765 (8.7%)	286 (10%)	0.008	667 (8.5%)	251 (10%)	0.011	41 (11%)	22 (20%)	0.014
**Fibromyalgia**	341 (3.9%)	115 (4.2%)	0.5	305 (3.9%)	98 (4.0%)	0.8	20 (5.3%)	7 (6.3%)	0.7
**Headache (incl. migraine)**	1,527 (17%)	474 (17%)	0.8	1,342 (17%)	422 (17%)	>0.9	84 (22%)	22 (20%)	0.6
**Headache (excl. migraine)**	1,031 (12%)	308 (11%)	0.4	900 (12%)	273 (11%)	0.6	62 (16%)	17 (15%)	0.8
**Osteoarthritis**	1,778 (20%)	645 (23%)	<0.001	1,613 (21%)	593 (24%)	<0.001	75 (20%)	26 (23%)	0.4
**Non-traumatic joint pain**	2,948 (34%)	901 (33%)	0.4	2,576 (33%)	810 (33%)	>0.9	145 (39%)	34 (31%)	0.13
**Surgery Type**			0.061			0.5			>0.9
**Major**	8,179 (97%)	2,590 (98%)		7,285 (97%)	2,305 (98%)		107 (98%)	217 (97%)	
**% Missing**	**3.9**	**3.8**		**4.0**	**4.1**		**4.0**	**1.8**	
**New persistent opioid use (outcome)**	1,121 (13%)	307 (11%)	0.024	1,023 (13%)	275 (11%)	0.014	52 (14%)	10 (9.0%)	0.2

Supplement 3: Refit Claims Cohort Characteristics Train Test Split

1 Statistics presented: median (IQR) for continuous variables; n (%) for categorical variables; education variables are census-tract proportions, presented as median (IQR)

2 Statistical tests performed: Wilcoxon rank-sum test; chi-square test of independence

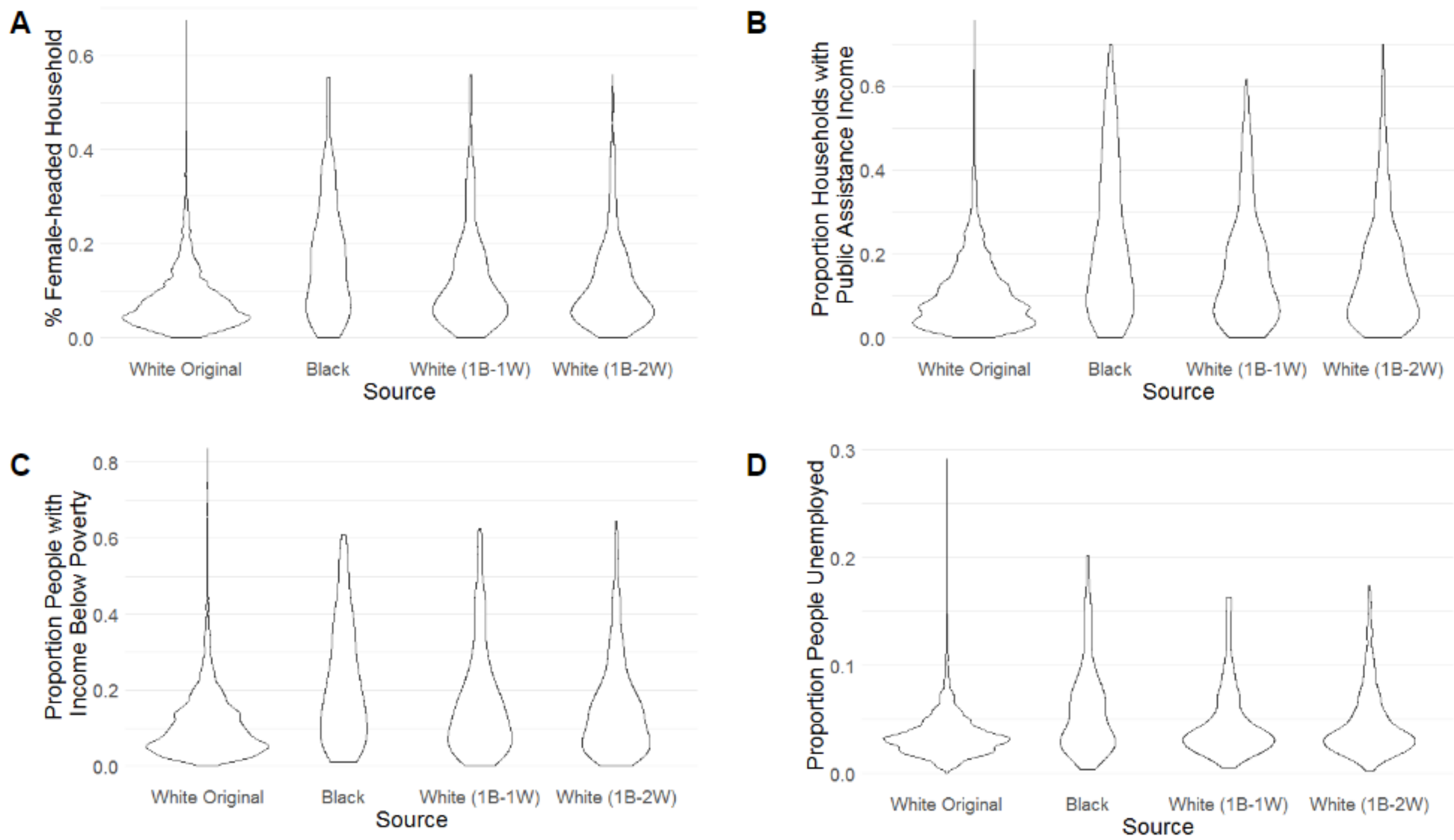

Supplement 4: Socioeconomic Variable Distributions for MGI Cohort that Comprise Disadvantage

Age, years	% with less than a high school degree
Female	Charlson Comorbidity Index
Preoperative opioid fills	Preoperative benzo use
Elixhauser Comorbidity Index	Lamotrigine fills
Average pain in week not at surgical site	Abdominal pain
PROMIS physical 4-item score	Arthritis joint pain
Chronic pain	Back pain
Perceived stress scale	Fibromyalgia
Life satisfaction	Headache
Hydrocodone/ acetaminophen # prescriptions	Headache, including migraine
Tramadol # prescription	Osteoarthritis
Smoking status	Surgery type

Supplement 5: Full Variable Table Used for Matching

Source	White Original Test[1] N = 5,181	White (1B-1W) Test[1] N = 269	White (1B-2W) Test[1] N = 538	Black Original Test[1] N = 269
**Age, year**	58 (45, 67)	51 (37, 62)	52 (37, 62)	50 (40, 61)
**Female**	2,636 (51%)	141 (52%)	282 (52%)	140 (52%)
**Opioid-naive**	2,454 (47%)	125 (46%)	243 (45%)	111 (41%)
**Elixhauser Comorbidity Index**	3 (1, 5)	4.0 (2.0, 7.0)	4.0 (2.0, 7.0)	4 (2, 6)
**Average pain in week not at surgical site**	2 (0, 4)	3.07 (1.00, 5.26)	3.02 (1.00, 5.00)	3 (0, 6)
% Missing	12	0	0	16
**PROMIS physical 4-item score**	18 (12, 20)	16.5 (12.0, 20.0)	16.7 (12.0, 20.0)	17 (12, 20)
% Missing	14	0	0	24
**Chronic pain**	2,944 (57%)	174 (65%)	353 (66%)	171 (64%)
% Missing	<0.1	0	0	0
**Perceived stress scale**	5 (2, 8)	5 (3, 7.2)	5 (3, 7)	5 (2, 8)
% Missing	16	0	0	25
**Life satisfaction**	7 (5, 9)	6.64 (5.0, 9.0)	6.75 (5.00, 9.00)	8 (5, 9)
% Missing	14	0	0	25
**Hydrocodone/ acetaminophen # prescription**	0 (0, 1)	0 (0, 1)	0 (0, 1)	0 (0, 1)
**Tramadol # prescription**	0 (0, 0)	0 (0, 0)	0 (0, 0)	0 (0, 0)
**Persistent opioid use (outcome)**	963 (19%)	64 (24%)	125 (23%)	58 (22%)

Supplement 6: MGI Matched Test Cohort Characteristics

1 Statistics presented: median (IQR) for continuous variables; n (%) for categorical variables

Characteristic	White Original[1] N = 2,454	White (1B-1W)[1] N = 111	White (1B-2W)[1] N = 222	Black Original[1] N = 111
**Age, years**	57 (42, 67)	47 (29, 61)	48 (32, 60)	48 (36, 60)
**Female**	1,229 (50%)	56 (50%)	115 (52%)	64 (58%)
**Smoking status**				
**Former**	781 (32%)	25 (23%)	54 (24%)	29 (26%)
**Never**	1,437 (59%)	68 (61%)	137 (62%)	70 (63%)
**Smoker**	226 (9.2%)	16 (14%)	29 (13%)	11 (9.9%)
**Unknown**	10 (0.4%)	2 (1.8%)	2 (0.9%)	1 (0.9%)
**Education**				
**Less than a high school degree**	0.06 (0.03, 0.10)	0.09 (0.05, 0.13)	0.09 (0.05, 0.14)	0.09 (0.05, 0.14)
**High school degree and/or some college**	0.62 (0.44, 0.71)	0.64 (0.53, 0.71)	0.62 (0.51, 0.70)	0.62 (0.49, 0.70)
**Bachelor's degree or higher**	0.31 (0.19, 0.51)	0.26 (0.15, 0.40)	0.27 (0.16, 0.43)	0.26 (0.15, 0.45)
% Missing	8.7	16	10	8.1
**Charlson Comorbidity Index**	3 (1, 7)	2.0 (0.5, 4)	2 (0, 5)	2 (0, 5)
**Drugs**				
**Preoperative benzo use**	361 (15%)	8 (7.2%)	17 (7.7%)	9 (8.1%)
**Lamotrigine fills**	0 (0, 0)	0 (0, 0)	0 (0, 0)	0 (0, 0)
% Missing	<0.1	0	0	0
**Diagnoses**				
**Abdominal pain**	508 (21%)	31 (28%)	70 (32%)	33 (30%)
**Arthritis joint pain**	1,114 (45%)	55 (50%)	110 (50%)	51 (46%)
**Back pain**	251 (10%)	18 (16%)	43 (19%)	22 (20%)
**Fibromyalgia**	98 (4.0%)	8 (7.2%)	15 (6.8%)	7 (6.3%)
**Headache (incl. migraine)**	422 (17%)	19 (17%)	40 (18%)	22 (20%)
**Headache (excl. migraine)**	273 (11%)	16 (14%)	27 (12%)	17 (15%)
**Osteoarthritis**	593 (24%)	19 (17%)	48 (22%)	26 (23%)
**Non-traumatic joint pain**	810 (33%)	34 (31%)	73 (33%)	34 (31%)
**Surgery Type**				
**Major**	2,305 (98%)	105 (100%)	207 (98%)	39 (98%)
% Missing	<0.1	5.4	5.0	1.8
**New persistent opioid use (outcome)**	275 (11%)	13 (12%)	22 (9.9%)	10 (9.0%)

Supplement 7: Refit Claims Matched Test Cohort Characteristics

1 Statistics presented: median (IQR) for continuous variables; n (%) for categorical variables; education variables are census-tract proportions, presented as median (IQR)

A

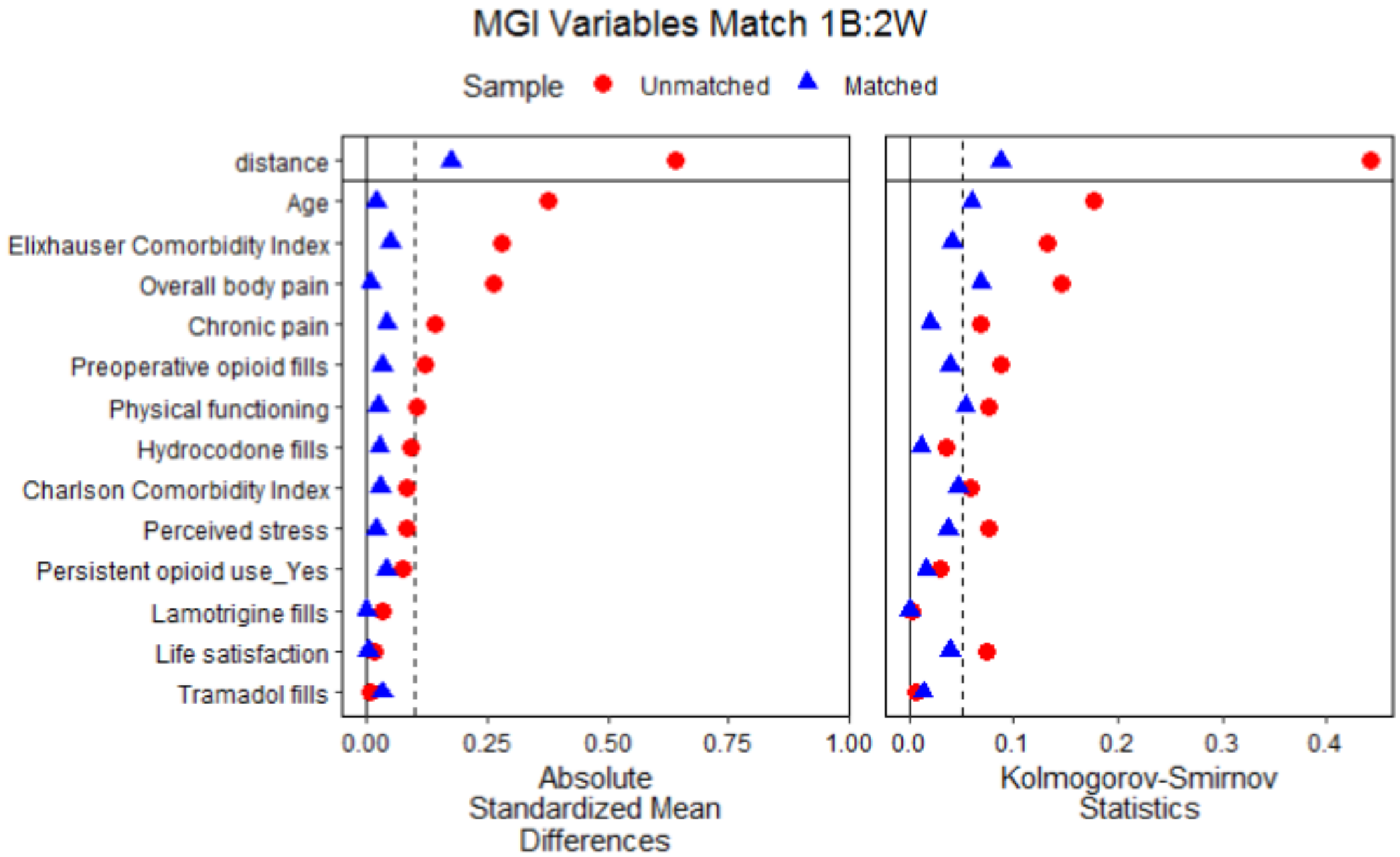

B

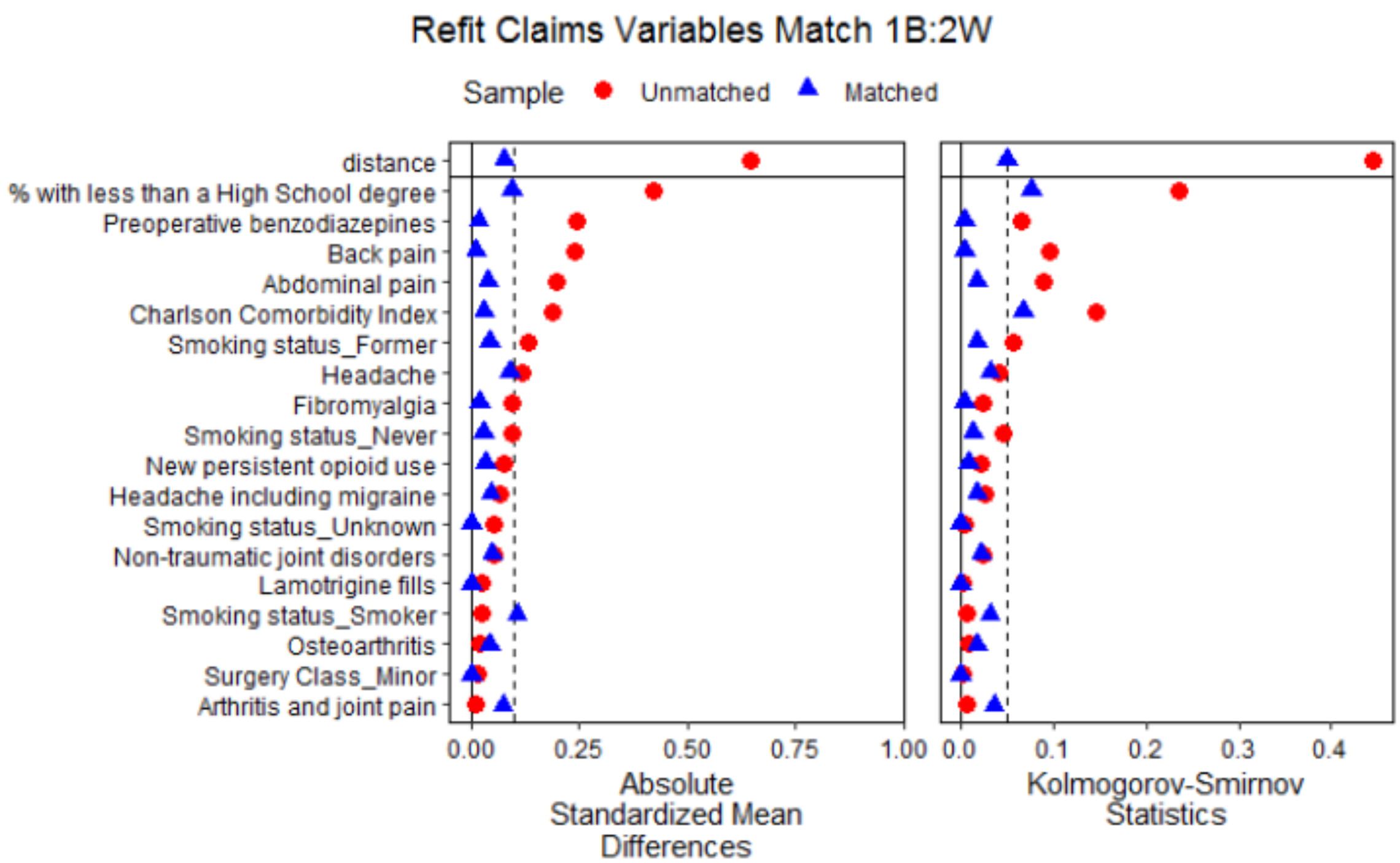

Supplement 8: Love Plots for Match Ratio 1 Black 2 White Covariates

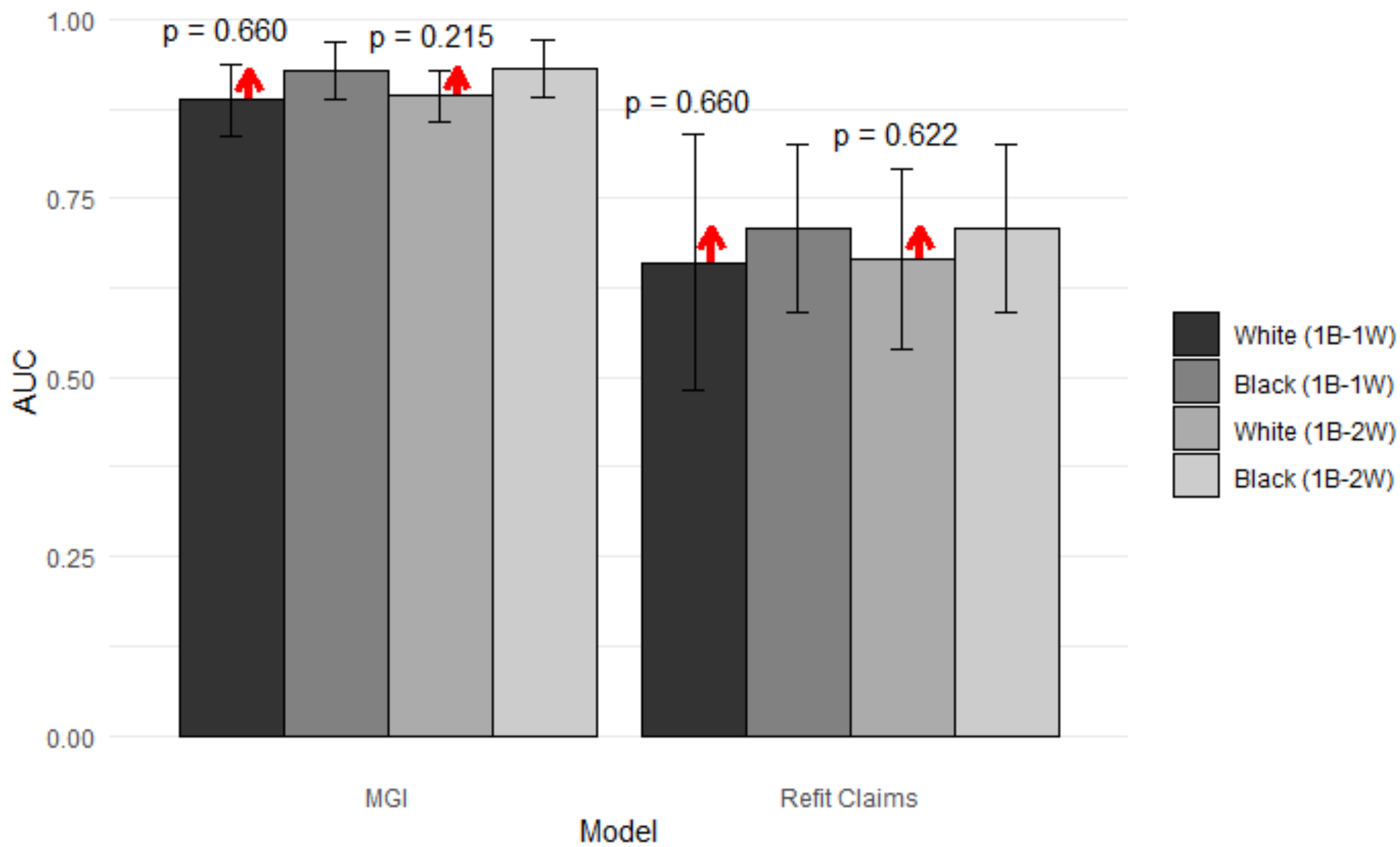

Supplement 9: Model Trained on Matched Cohort Performance Tested on Respective Matched Cohort

Characteristic	White	Black	Matched White (1B:2W)
AUC	0.860	0.926	0.891
Sensitivity	0.789	0.897	0.864
Specificity	0.788	0.763	0.738
PPV	0.460	0.510	0.500
NPV	0.942	0.964	0.947
MCC	0.482	0.559	0.519
Proportion of Positives	0.319	0.379	0.401

Supplement 10: MGI Model Performance Metrics Across Racial Subgroups
